# Energy deficit and hospital length of stay can be reduced by quality management of nutrition therapy: the ICU dietitian is essential

**DOI:** 10.1186/cc9796

**Published:** 2011-03-11

**Authors:** L Soguel, JP Revelly, C Longchamp, MD Schaller, MM Berger

**Affiliations:** 1 HES-SO, Geneva, Switzerland; 2 CHUV, Lausanne, Switzerland

## Introduction

Several studies show that nutrition delivery is insufficient, resulting in large energy deficits during the ICU stay [1]: the problem persists despite the diffusion of guidelines. The barriers to guideline implementation are known [2]. This study aimed at measuring the clinical impact of a two-step interdisciplinary quality nutrition program incorporating knowledge of the barriers.

## Methods

A prospective interventional study over three periods (A: baseline, B and C: intervention periods) in the mixed ICU of a university teaching hospital. Inclusion: patients requiring >72 hours of ICU. Intervention was a two-step quality program after baseline analysis: first, implementation of feeding guidelines; and second, additional presence of an ICU dietitian. Variables: anthropometry, severity scores, energy delivery and balances (daily, day 7, discharge), feeding route, length of stay, and mortality.

## Results

In total, 604 admissions and 6,073 days were analyzed. Patients in period A were less sick (lower SAPS and less rapidly fatal McCabe scores) than those of periods B and C. Energy delivery and balance increased gradually: impact was particularly marked in the cumulated energy balance on day 7 (*P *< 0.001). The feeding technique changed: use of EN increased from A to B (stable in C); combined and PN increased progressively. Oral intakes were uniformly low (305 kcal/day). Hospital mortality paralleled severity in periods B and C. The hospital stay was shorter in period C (*P *= 0.048). See Table 1.

**Table 1:** 

	Period A: baseline	Period B: new protocol	Period C: protocol + dietitian	*P *value
Cumulated energy balance day 7	-5,870 ± 3,314	-5,307 ± 3,131	-3,946 ± 3,682*	< 0.001
Discharge energy balance	-6,972 ± 4,994	-5,996 ± 3,711*	-5,380 ± 4,998*	0.002
Energy delivery (kcal/kg/day)	14.8 ± 12.8	17.1 ± 12.7*	17.8 ± 12.6*	< 0.0001

* Significant *post hoc *difference.

## Conclusions

A bottom-up protocol improved nutritional support. The ICU dietitian further improved the process (early introduction, feeding route), achieving better early energy balance.
